# Downregulation of CCR5 on brain perivascular macrophages in simian immunodeficiency virus‐infected rhesus macaques

**DOI:** 10.1002/brb3.3126

**Published:** 2023-06-27

**Authors:** Julian B. Hattler, Derek L. Irons, Jiangtao Luo, Woong‐Ki Kim

**Affiliations:** ^1^ Department of Microbiology and Molecular Cell Biology Eastern Virginia Medical School Norfolk Virginia USA; ^2^ Department of Health Systems and Population Health Sciences Tilman J. Fertitta Family College of Medicine, University of Houston Houston Texas USA; ^3^ Division of Microbiology Tulane National Primate Research Center Covington Louisiana USA; ^4^ Department of Microbiology and Immunology Tulane University School of Medicine New Orleans Louisiana USA

**Keywords:** CCR5, CD8 T lymphocytes, endocytosis, HIV, perivascular macrophages, SIV

## Abstract

**Background:**

C‐C chemokine receptor 5 (CCR5) is a major coreceptor for Human immunodeficiency virus (HIV) and simian immunodeficiency virus (SIV) cell entry; however, its role in brain pathogenesis is largely understudied. Thus, we sought to examine cell type‐specific protein expression of CCR5 during SIV infection of the brain.

**Methods:**

We examined occipital cortical tissue from uninfected rhesus macaques and SIV‐infected animals with or without encephalitis using immunohistochemistry and immunofluorescence microscopy to determine the number and distribution of CCR5‐positive cells.

**Results:**

An increase in the number of CCR5+ cells in the brain of SIV‐infected animals with encephalitis was accounted for by increased CD3+CD8+ cells expressing CCR5, but not by increased CCR5+ microglia or perivascular macrophages (PVMs), and a concurrent decrease in the percentage of CCR5+ PVMs was observed. Levels of CCR5 and SIV Gag p28 protein expression were examined on a per‐cell basis, and a significant, negative relationship was established indicating decreased CCR5 expression in productively infected cells. While investigating the endocytosis‐mediated CCR5 internalization as a mechanism for CCR5 downregulation, we found that phospho‐ERK1/2, an indicator of clathrin‐mediated endocytosis, was colocalized with infected PVMs and that macrophages from infected animals showed significantly increased expression of clathrin heavy chain 1.

**Conclusions:**

These findings show a shift in CCR5‐positive cell types in the brain during SIV pathogenesis with an increase in the number of CCR5+ CD8 T cells, and downregulated CCR5 expression on infected PVMs, likely through ERK1/2‐driven, clathrin‐mediated endocytosis.

## Graphical abstract

In this study, we provide in vivo evidence that SIV infection downregulates CCR5, one of the two major HIV/SIV coreceptors, in the brain of rhesus macaques, likely through clathrin‐mediated endocytosis. This is a clinically relevant information, which explains why CCR5 inhibition prevents new infections, but is not effective at suppressing viral replication from cells that have been already infected.

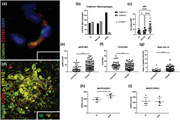

## 1 INTRODUCTION

Human immunodeficiency virus (HIV) infection spreads through the body at a very high rate, with diverse sites of infection established in the first 3 days, including immune privileged sites such as the brain. Studies examining early antiretroviral therapy (ART) starting as early as 3 days post infection have shown that this rapid dissemination results in seeding of viral reservoirs that persist through long‐term ART treatment (Whitney et al., 2014). With the inability to eliminate these reservoirs through current ART regimens, it has become vitally important to understand the cells that make up these reservoirs and their role in HIV pathogenesis (Hsu et al., 2018; Ko et al., 2019).

Previous studies by our group have shown brain perivascular macrophages (PVMs) and microglia to be the major viral reservoir in the brain (Ko et al., 2019). These cells are infected with macrophage‐tropic HIV/simian immunodeficiency virus (SIV) through CD4 receptor and a coreceptor C‐C chemokine receptor 5 (CCR5) (Edinger et al., 1997). Viral envelope protein gp120 binds strongly with CD4 causing a conformational change in the gp120 protein which then allows for CCR5 interaction, leading to membrane fusion and viral entry into the cell (Cormier et al., 2000; Tamamis & Floudas, 2014). With low expression of CD4 on macrophages, including PVMs, levels of CCR5 surface expression likely contribute to the susceptibility of these cells to HIV infection. Interestingly, a mutation in CCR5, CCR5∆32, truncates the receptor leading to lower membrane expression and degrees of resistance to infection both in vitro and clinically in two successful ∆32/∆32 bone marrow transplantations in which the patients showed no viral rebound despite cessation of ART (Hütter et al., 2009). CCR5 is a seven‐transmembrane G protein‐coupled receptor expressed on immune cells, which facilitates the trafficking of cells during the inflammatory response. In addition, CCR5 has been shown to play a role in activation of CD8 T cells, with its expression highly correlated with activation status (Wang et al., 2019). Ligands for this receptor include CCL3, CCL4, CCL3L1, and CCL5 (RANTES), all of which, upon binding, cause rapid internalization of ligand and receptor (Venuti et al., 2017, 2015). In vitro work examining this internalization has shown the presence of CCR5 in invaginated clathrin pits following CCL5 treatment, as well as CCR5 internalization inhibited by depletion of clathrin (Mueller & Strange, 2004; Signoret et al., 2005). However, reports differ on the fate of CCR5 following internalization, some showing quick recycling to the cell surface, while others demonstrate sustained downregulation (Mueller et al., 2002; Signoret et al., 2000; Venuti et al., 2015). This may suggest multiple, cell‐fate specific, signaling pathways regulating CCR5 surface expression. ERK1/2 signaling has been shown to both colocalize with internalized CCR5 and prevent internalization of CCR5, when inhibited (Venuti et al., 2015). Further, the results of Venuti et al. (2015) indicate receptor degradation, showing cell surface re‐expression dependent upon de novo synthesis.

The importance of CCR5 in the infection of myeloid cells is well established; however, its presence and role in HIV brain pathogenesis is still unknown, in part due to the paucity of specific anti‐human CCR5 antibodies that are effective in routinely processed—that is, formalin‐fixed, paraffin‐embedded—tissue. For this study, we tested multiple commercially available CCR5 antibodies, validating a single antibody, as well as a monoclonal human CCR5 antibody kindly gifted by Dr. Mathias Mack at Regensburg University (Regensburg, Germany) for immunohistochemical reactivity to human and monkey formalin‐fixed tissue. We showed an increase in CCR5‐positive, activated CD8 infiltrates driving an increase in total CCR5‐positive cells in the brains of encephalitic animals. Despite this progression to severe infection, there was a decline in percentage of CCR5‐positive PVMs, which was surprising considering myeloid cell‐driven neuropathogenesis in HIV/SIV infection. We showed strong co‐localization of clathrin heavy chain 1 with PVMs, a significant inverse association between SIV Gag p28 protein and CCR5 levels, and increased levels of phospho‐ERK1/2, indicating clathrin‐mediated endocytosis, degradation, and sustained downregulation of CCR5 in SIV‐infected PVMs.

## 2 MATERIALS AND METHODS

### 2.1 Animals

Archival sections from a total of 23 rhesus macaque (*Macaca mulatta)*, 14 adult and nine neonates listed in Table 1, were used in this study. All animals except those marked with an asterisk (*) were housed at the Tulane National Primate Research Center (TNPRC), and all procedures were approved by the Tulane University Institutional Animal Care and Use Committee. Animals marked with an asterisk in Table 1 were housed at the New England Regional Primate Research Center (NERPRC) ,and all procedures were approved by the Harvard University Institutional Animal Care and Use Committee. All procedures were in accordance with the Guide for the Care and Use of Laboratory Animals and recommendations of the Weatherall report and performed under the direction of veterinarians. Animals were divided into groups based on age and infection status, with all the infected animals inoculated with intravenous SIVmac251. Following sodium pentobarbital euthanasia, tissue was formalin fixed and paraffin embedded (FFPE). Five‐micrometer thick FFPE sections were used for this study. SIVE animals were defined by the presence of SIV Gag proteins, accumulation of macrophages, and the presence of multinucleated giant cells in the brain.

**TABLE 1:** Animals used in this study.

Animal ID	Age (years) at euthanasia and sex	Length (years) of Infection	Infection status at euthanasia
11A014	4.77 M	N.A.	Uninfected
11A023	4.69 M	N.A.	Uninfected
11A313	5.02 M	N.A.	Uninfected
11A314	8.99 M	N.A.	Uninfected
11A635	5.14 M	N.A.	Uninfected
11A563	8.19 M	0.19	SIVnoE
11A837	8.28 M	0.61	SIVnoE
12A305	7.95 M	0.97	SIVnoE
12A500	5.42 M	1.26	SIVnoE
12A544	6.63 M	1.33	SIVnoE
10A067	5.70 M	0.46	SIVE
11A554	10.28 M	0.27	SIVE
A07‐136*	3.67 M	Unknown	SIVE
A07‐237*	3.48 M	0.5	SIVE
11A472	0.01 M	N.A.	Uninfected
11A527	0.01 F	N.A.	Uninfected
12A006	0.52 F	N.A.	Uninfected
10A718	0.57 F	0.22	SIVnoE
11A489	0.21 F	0.021	SIVnoE
11A490	0.10 M	0.1	SIVnoE
11A521	1.04 F	1.04	SIVnoE
12A005	0.23 M	0.23	SIVnoE
12A611	0.18 F	0.17	SIVnoE

### 2.2 Human samples

The Manhattan HIV Brain Bank (MHBB) graciously provided FFPE sections of frontal white matter for 3 HIVE cases and 3 seronegative controls (Table 2). Controls had minimal non‐diagnostic abnormalities when autopsied. Postmortem intervals were all less than 48 h.

**TABLE 2:** De‐identified human subjects characteristics.

Project ID	Age (years) at death and sex	Infection status at death
01640	63 M	Uninfected
01819	65 M	Uninfected
01923	61 M	Uninfected
01555	47 M	HIVE
01580	50 M	HIVE
02390	57 F	HIVE

### 2.3 Immunohistochemistry

Immunohistochemistry was performed as previously described (Filipowicz et al., 2016). In brief, deparaffinization and rehydration were performed, and sections were treated with antigen‐retrieval solution, citrate or tris‐based antigen unmasking solution (Vector Laboratories, Burlingame, CA, USA). The sections were then washed and blocked for endogenous peroxidase activity (Bloxall; Vector Laboratories). The sections were blocked using 5% normal goat or horse serum for 30 min and then incubated with primary antibodies (Table 3) at room temperature for 1 h or overnight at 4°C. Biotinylated secondary antibodies were used for 30 min. The sections were subsequently incubated with Avidin‐Biotin‐Peroxidase Complex (Vectastain Elite ABC kit; Vector Laboratories), developed with diaminobenzidine (DAB; Agilent Dako, Santa Clara, CA, USA) for 10 min, and counterstained with hematoxylin nuclear counterstain. Finally, the sections were dehydrated and mounted using VectaMount (Vector Laboratories).

**TABLE 3:** Antibodies used in this study.

Antigen	Clone	Isotype	Manufacturer	IHC Conc.	IF Conc.	Species tested
CCR5		Rabbit IgG	Proteintech	1:800	1:600	Macaque
CCR5	MC‐5	Mouse IgG2a	Matthias Mack	1:100	1:20	Human
Iba1		Goat IgG	Abcam		1:400	Macaque
CD163	EDHu‐1	Mouse IgG1	Bio‐Rad		1:50	Both
TMEM119	pT202/pY204.22A	Rabbit IgG	Abcam		1:20	Human
CD3	CD3‐12	Rat IgG	Abcam	1:250	1:50	Both
p‐ERK1/2		Mouse IgG1_k_	Santa Cruz		1:1,000	Macaque
CHC17	9M2G2	Rabbit IgG	Novus		1:50	Macaque
SIV p28*	3F7	Mouse IgG1_k_	Fitzgerald		1:100	Macaque

Abbreviation: IHC, immunohistochemistry.

* FITC pre‐conjugate antibody.

### 2.4 Immunofluorescence

Sections were deparaffinized, rehydrated, and treated with antigen retrieval as stated above. The sections were washed in phosphate‐buffered saline with 0.2% fish skin gelatin (Sigma–Aldrich, St. Louis, MO, USA) permeabilized in wash buffer with 0.1% Triton X‐100, washed, and then blocked with 5% normal horse or goat serum. Without a wash, the sections were incubated with primary antibodies (Table 3) at room temperature for 1 h or 4°C overnight. The sections were then washed and incubated with Alexa fluor 488‐, 594‐, or 647‐, and Cy3‐conjugated secondary antibodies for 1 h. All primary and secondary antibodies were individually applied with the matched secondary following its primary counterpart. Where possible, DAPI (4',6–diamidino–2–phenylindole) nuclear stain was used. Autofluorescence was quenched with 10 mM CuSO_4_ solution in 50 mM ammonium acetate buffer for 45 min, rinsed in water, and then mounted using Aqua‐Mount Mounting Medium (Thermo Scientific, Waltham, MA, USA). The sections were visualized using a ZEISS Axio Observer.Z1 fluorescence microscope (Jena, Germany) or ZEISS LSM 880 confocal laser scanning microscope.

### 2.5 Immunohistochemistry image analysis

Semi‐quantitative analysis was performed; 15 (CD3) or 21 (CCR5) images were acquired per section at a magnification of 20× on a Nikon Coolscope digital microscope and manually counted. Counts were recorded as total positive cells per 15 or 20 images for each animal or human subject. Statistical analysis between groups was performed in GraphPad Prism 9.0.

### 2.6 Immunofluorescence image analysis

Images were acquired on a ZEISS Axio Observer.Z1 fluorescence microscope at a magnification of 20× or 40× using ZEISS AxioVision Release 4.8.2 or Zen blue software, or on a ZEISS LSM 880 confocal laser scanning microscope using a 63× immersion oil objective and the Zen Black software. Exposure was adjusted and kept consistent within each quantification. Background subtraction was performed where possible, and background mean pixel intensity (MPI) per image was calculated and subtracted to calculate normalized MPI. For phospho‐ERK1/2 staining, background subtraction was not applied, and data were calculated as MPI. Percent CCR5‐positive CD3 or CD163 cells, respectively, were calculated by randomly counting fifty total cells as positive or negative and calculating percentage positive per animal. Statistical analysis between groups was performed in GraphPad Prism 9.0.

### 2.7 NanoString

Total RNA was extracted from FFPE tissue samples from uninfected (UI) and SIVE macaques using Qiagen FFPE RNeasy Kit following manufacturer's instructions with one modification, lowering elution volume to 10 μL to concentrate sample. Three hundred nanograms of total RNA per samples, as per NanoString recommendations for analysis of RNA extracted from FFPE tissue, was probed using the nCounter NHP Immunology V2 CodeSet on a NanoString nCounter Flex, and data were normalized using nSovler 4.0 before transferring to GraphPad Prism 9.0 for statistical analysis. Degree of RNA degradation was assessed using a Thermo Scientific NanoDrop 1000, with all samples meeting NanoString recommendations.

### 2.8 Statistical analysis

Statistical analysis was performed using GraphPad Prism version 9.3.1, and statistical tests used are indicated in figure legends, with the exception of the following. A Ph.D. biostatistician (J.L.) generated a generalized additive model to fit the relationship between CCR5 expression and SIV Gag p28 (Ni et al., 2018). The model includes two parts. Both linear and spline parts of SIV p28 are statistically significantly associated with CCR5 expression with *p*‐values < .0001. Our conclusion is that CCR5 expression and SIV p28 are statistically significantly associated via a generalized additive model, but not in a simple way.

## 3 RESULTS

### 3.1 Cells expressing HIV coreceptor CCR5 increase with progression to AIDS and encephalitis

**FIGURE 1:**
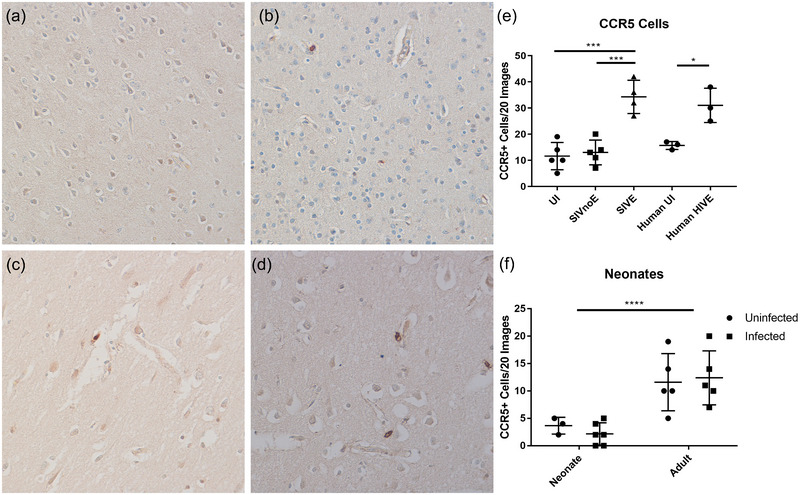
C‐C chemokine receptor 5 (CCR5) increases with infection. Immunohistochemistry (IHC) staining for CCR5 was performed on rhesus macaque brain occipital cortices. Representative figures of neonate simian immunodeficiency virus (SIV) infected (a), adult uninfected (b), adult SIVnoE (c), and adult SIVE (d) are shown here with brown showing positive diaminobenzidine (DAB) staining and blue/purple showing hematoxylin nuclear counterstain. There is a significant increase in the number of CCR5+ cells in encephalitic animals, one‐way analysis of variance (ANOVA) with Tukey's multiple comparison test, *p*‐values = .0002 and .0003, and humans, unpaired *t* test *p*‐value = .0169 (e). Neonates had significantly less CCR5‐positive staining than adults; however, there was no change with infection, two‐way ANOVA with row factor *p*‐value = .0002 (f).

Due to the critical role played by CCR5 in HIV infection of macrophages, we hypothesized that the presence of CCR5 on brain PVMs, the major cellular target of HIV/SIV in the brain, would increase throughout disease progression. To begin investigating CCR5 in the brain, we examined occipital cortical tissue from adult rhesus macaques at different stages of infection, UI animals, SIV‐infected animals with no signs of AIDS and encephalitis (SIVnoE), and SIV‐infected, AIDS animals with encephalitis (SIVE), through immunohistochemistry. Surprisingly, SIVnoE animals showed no significant difference from UI controls in total number of CCR5‐positive cells. However, SIVE animals had significantly more CCR5‐positive cells than UI and SIVnoE animals, showing an increase in CCR5+ cells with progression to AIDS and severe disease (Figure 1b–e). This finding was recapitulated in postmortem human samples; patients with HIVE also showed significantly more CCR5‐positive cells than seronegative subjects. Postmortem tissue from non‐ART‐treated, non‐encephalitic, but HIV‐infected patients was not available.

Unlike adults, HIV‐infected infants and neonatally SIV‐infected macaques do not develop encephalitis, despite rapid disease progression and neurocognitive changes (Bohannon et al., 2020; Delery et al., 2019; Dickson et al., 1993; Kure et al., 1991). As such we compared UI and SIV‐infected infant rhesus macaques for CCR5 expression to elucidate whether differential expression of the HIV/SIV coreceptor may play a role in lesion formation. No change was found between UI and infected neonates in the number of CCR5‐positive cells (Figure 1f); however, numbers were significantly lower in infants than adults as reported by Delery et al. (2019). Due to extremely low CCR5 expression in uninfected and infected neonates/infants and the complexity of macrophage subsets in neonatal SIV infection, neonates were not investigated further in this study (Bohannon et al., 2020).

### 3.2 Microglia, T cells, and PVMs express CCR5

**FIGURE 2:**
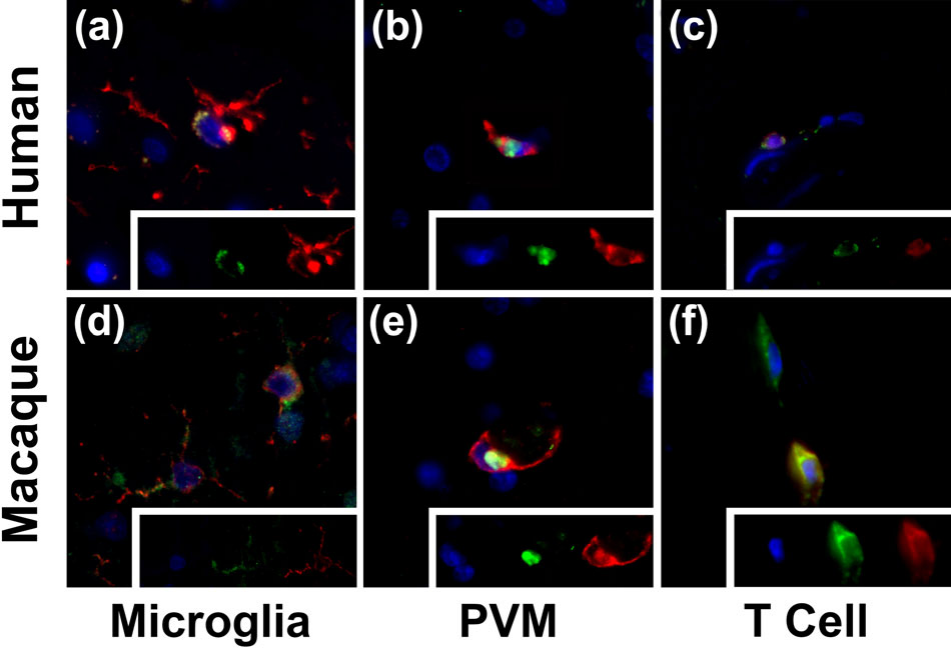
T cells, microglia, and perivascular macrophages (PVMs) express C‐C chemokine receptor 5 (CCR5) in rhesus macaque and human brains. Triple‐label immunofluorescence staining was performed for CCR5 (green) and IBA‐1 in macaque and TMEM119 in human, CD163, or CD3 lineage markers (red), and DAPI nuclear stain (blue) showing CCR5 expression in microglia, PVMs, and T cells, respectively, in human (a–c) and macaque (d–f) tissues.

CCR5 expression on various cell types throughout the body has been reported, and as such we set out to examine the phenotype of cells expressing CCR5 in the brains of uninfected and infected macaques and humans. Using triple‐label immunofluorescence staining for CCR5, DAPI, and CD3, TMEM119/IBA‐1, and CD163 lineage markers, co‐expression with CCR5 was examined, with positive staining for all three cell types observed in both uninfected and infected tissues. IBA‐1 and TMEM119 microglia markers colocalized with CCR5 membrane staining in human (Figure 2a) and macaque (Figure 2d) cortical tissue, respectively. However, expression levels were visually lower in microglia than in other cell types, and CCR5+ microglia were found scattered with limited frequency in both human and macaque tissue. Staining for CD163 macrophage marker also colocalized with CCR5 in humans (Figure 2b) and macaques (Figure 2e). Interestingly, CD163+ PVMs exhibited both membrane and cytoplasmic CCR5 staining in both uninfected and infected brains (Figure 1). The strongest CCR5 staining was observed in colocalization with CD3‐positive cells, which have been shown to be almost exclusively CD8 and not CD4 T cells in the brains of SIV‐infected macaques, approximately 95% CD8 T cells (Figure 2c,f) (Hsu et al., 2018; Kim et al., 2004; Marcondes et al., 2001; Moniuszko et al., 2003). Despite the strong expression of CCR5, these cells cannot be infected by SIV without the CD4 receptor and therefore may not play a direct role in HIV infection and reservoir formation in the brain; however, CCR5 expression is rather a strong indicator of antigen‐specific activation on CD8 T cells.

### 3.3 Activated, brain‐infiltrating, CD8 T cells, not PVMs or microglia, account for the observed increase in CCR5+ cell count

**FIGURE 3:**
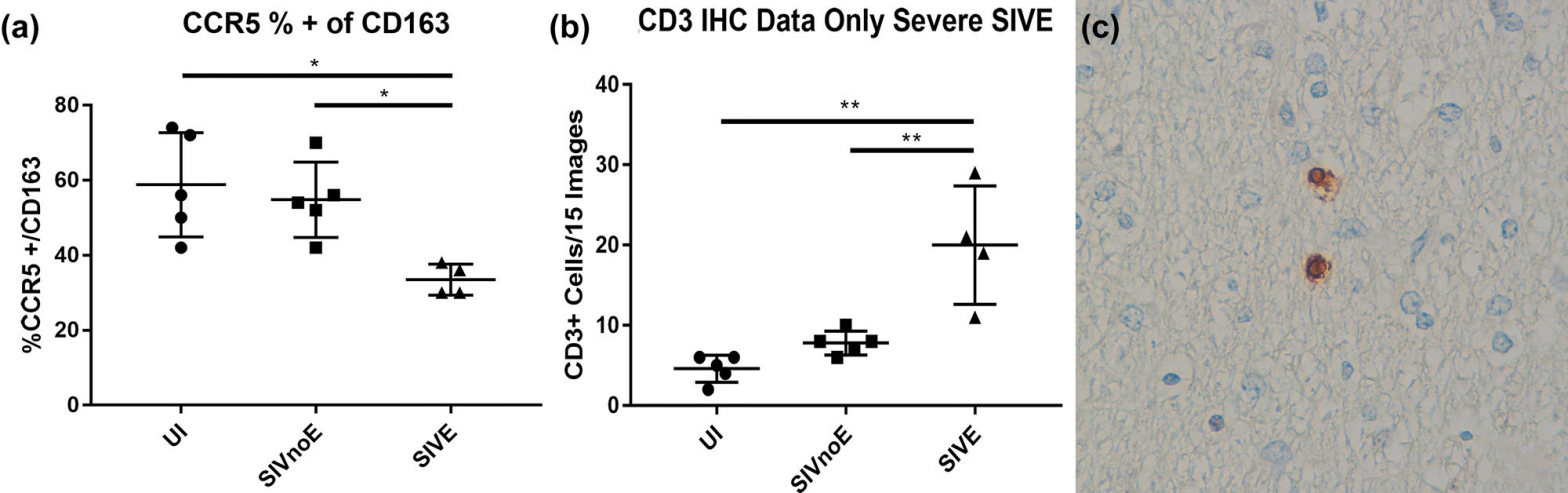
T cells and not perivascular macrophages (PVMs) or microglia account for the increase in the number of C‐C chemokine receptor 5 (CCR5)‐positive cells in the brain. Triple‐label immunofluorescence staining was performed for CCR5, CD163, and DAPI showing a significant decrease in the percentage of CD163+ PVMs that are positive for CCR5 in animals with encephalitis compared with both uninfected (UI) and SIVnoE, one‐way analysis of variance (ANOVA) with Tukey's multiple comparison test, *p*‐values = .0113 and .0297 (a). Immunohistochemistry (IHC) staining for CD3 was performed on UI, SIVnoE, and SIVE animals showing a significant increase in infiltrating CD3 T cells in animals with encephalitis, one‐way ANOVA with Tukey's multiple comparison test, *p*‐values = .0004 and .0026 (b and c).

Because PVMs are the major cell type infected with HIV and SIV in the brain, we assumed that the increase in CCR5‐positive cell counts would closely correlate with an increase in CCR5+ PVMs. We therefore examined the percentage of CCR5+ CD163 cells across stages of infection. Having observed no significant change in total number of CCR5‐positive cells between UI and SIVnoE groups (Figure 1e), it was not surprising to find that the percentage of CCR5+ PVMs in SIVnoE animals was not significantly different from that of uninfected animals. Contrary to our expectation, SIVE animals showed a decrease in percentage of CCR5+ PVMs (Figure 3a) in spite of the observed increase in total CCR5‐positive cells. In an attempt to explain this unexpected result, we examined the number of CD3 T cells across infection through immunohistochemistry. UI and SIVnoE showed no significant difference in number of CD3‐positive cells; however, there was a significant increase in CD3 cells in animals with encephalitis, with more cells in and around encephalitic lesions (Figure 3b,c). CD3 cells in UI and SIVnoE animals were found both in association with vessels and scattered in the brain parenchyma. To confirm these cells were positive for CCR5, contributing to the increase in total CCR5‐positive cells, triple‐label immunofluorescence was performed, and the percentage of CCR5+ CD3 cells was established with approximately 80% of CD3 T cells co‐expressing CCR5 in animals with encephalitis. Of note, both SIVnoE and SIVE animals showed significantly higher percentage of CCR5+ CD3 T cells than UI, with no significant variation in percentage between the two groups (Figure S2). Taken together, these results show that infiltrating, activated CCR5+ CD8 T cells and not PVMs account for the increase in total number of CCR5‐positive cells in the brains of encephalitic animals. CCR5+ microglia were rare and did not contribute to changes in subpopulation distribution of total CCR5‐positive cells.

### 3.4 Productively infected PVMs downregulate CCR5

**FIGURE 4:**
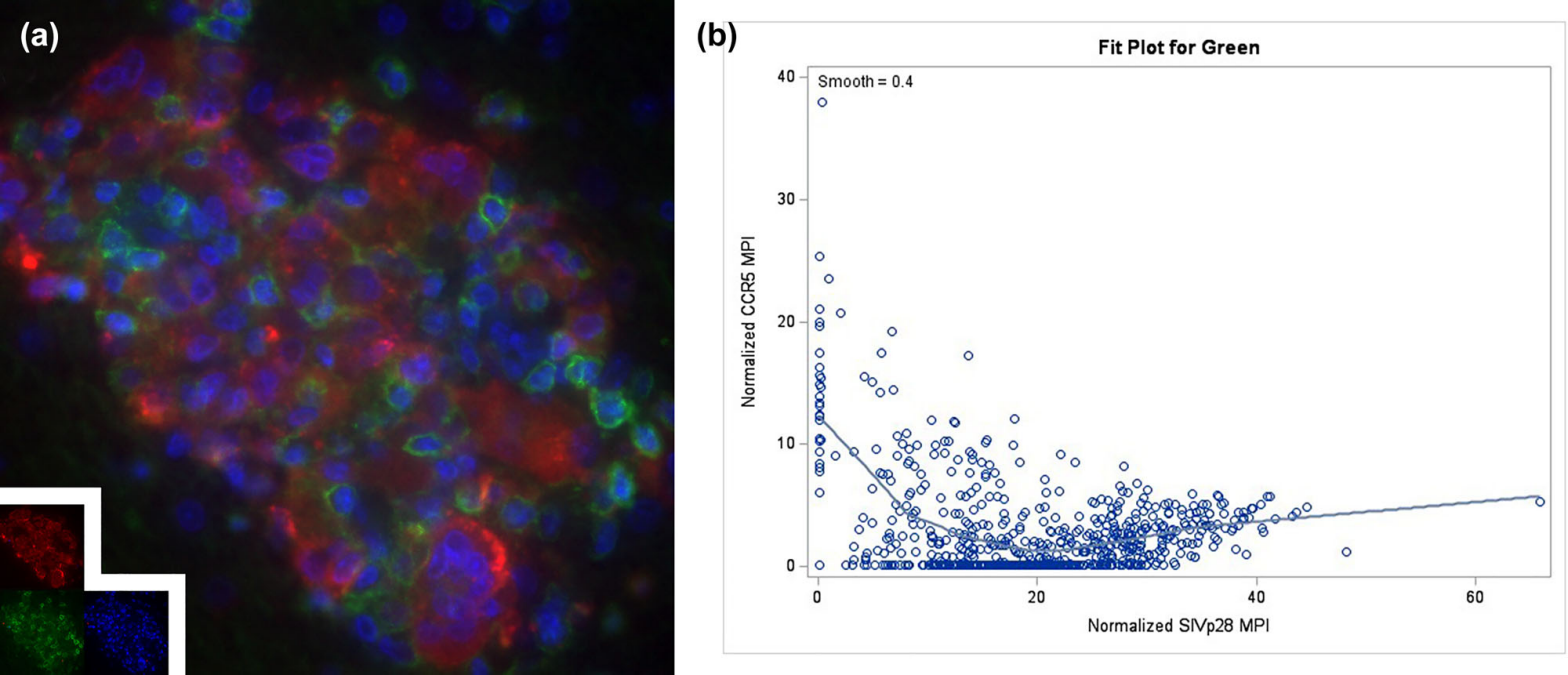
Simian immunodeficiency virus (SIV) p28 protein is associated with decreased CCR5 in SIV p28‐positive cells. Triple‐label immunofluorescence microscopy was performed for SIV p28 (red), CCR5 (green), and DAPI (blue) in the SIVE rhesus macaques, and images were acquired on a ZEISS AxioVision Release 4.8.2 (a). Background subtraction using the AxioVision and Adobe Photoshop software was applied. Normalized mean pixel intensity (MPI) data were sent to a statistician (Jiangtao Luo) who modeled the relationship between SIV p28 and CCR5 using a general additive model with a linear function followed by a smooth function estimated using a scatterplot smoother with both parts reaching statistical significance with *p*‐value < .0001 (b).

With increased severity of infection and increased numbers of infected PVMs in SIVE animals, (Filipowicz et al., 2016) we expected to see an increase in the percentage of CCR5+ PVMs but found the opposite. Given these observations, we updated our initial hypothesis and sought to investigate whether spreading infection may cause an internalization and downregulation, or complete degradation, of CCR5 following PVM infection, leading to a decreased percentage. To test this, we performed immunofluorescence staining for CCR5 and SIV Gag p28 protein, showing CCR5/SIV p28 colocalization in some PVMs of SIVE animals (Figure 4a). Normalized MPI was determined on a per‐cell basis for both CCR5 and SIV p28, in both SIV p28‐positive and SIV p28‐negative cells to determine the effect of infection on CCR5 expression. In an effort to avoid CCR5+ CD8 T cells, small round SIV p28‐negative cells were excluded from the analysis. A scatter (XY) plot of SIV p28 versus CCR5 MPI for each cell demonstrated an association between SIV p28 and CCR5 with increased SIV p28 significantly corresponding to lower expression of CCR5 (Figure 4b). CCR5 MPI demonstrated a range of expression at each corresponding expression levels of SIV p28, indicating a method of sustained downregulation that may or may not lead to complete absence of the receptor on the cell.

### 3.5 Activated ERK1/2 MAP kinases in association with downregulated CCR5 in SIV‐p28+ macrophages

**FIGURE 5:**
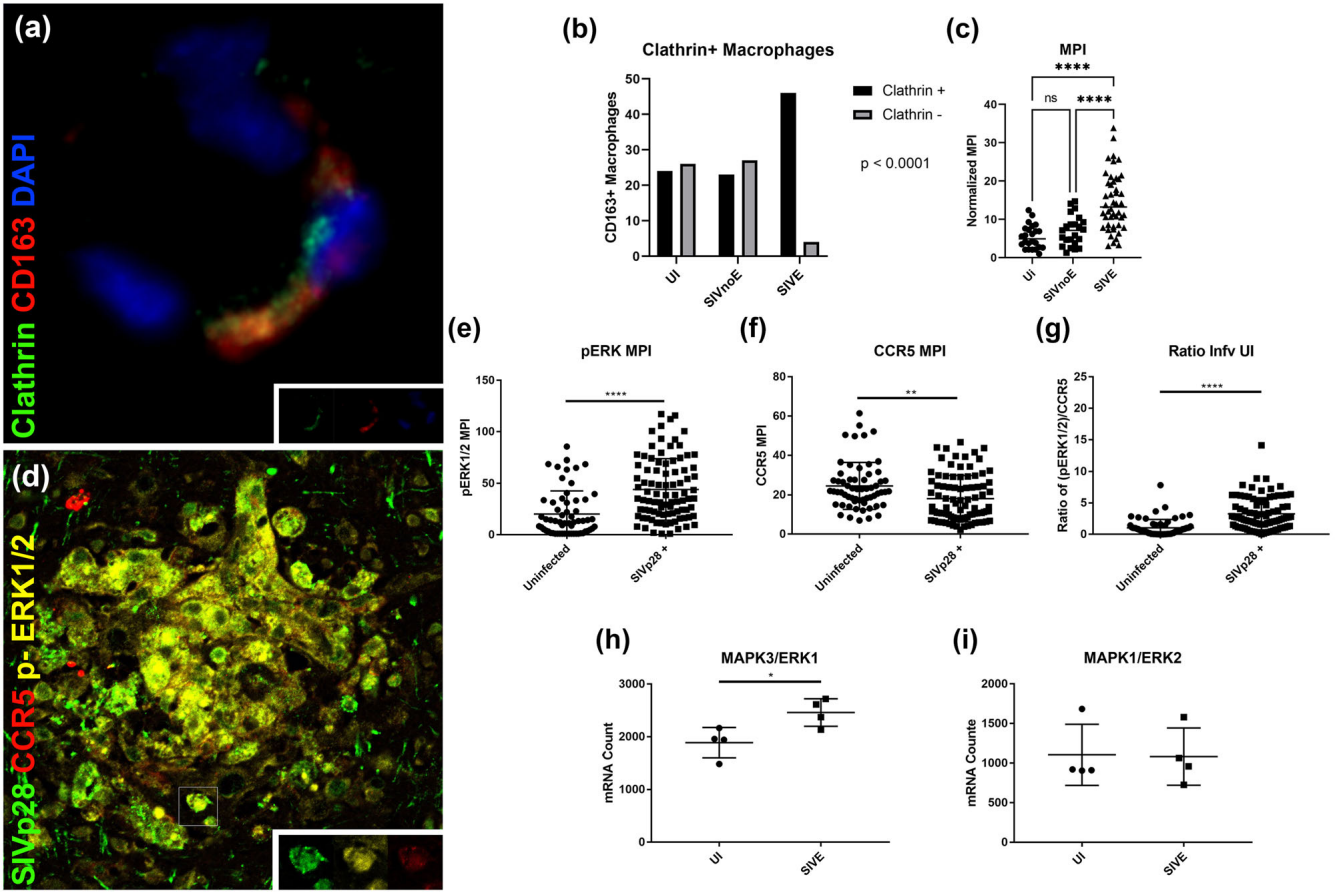
Phospho‐ERK1/2 is upregulated in infected cells. Triple‐label immunofluorescence staining for clathrin heavy chain 1 (green), CD163 (red), and DAPI (blue) was performed on SIVE tissue (a) showing colocalization of clathrin heavy chain 1 with perivascular macrophages (PVMs). Chi Squared test was performed comparing proportion of clathrin‐positive CD163+ macrophages between groups, with 50 macrophage cells analyzed per group (b). Mean pixel intensity (MPI) was measured and normalized for each clathrin‐positive macrophage, and a one‐way analysis of variance (ANOVA) with Tukey's post hoc test was performed to compare between groups showing significantly higher clathrin MPI in SIVE animals, *p*‐value < .0001 and < .0001 (c). Triple‐label immunofluorescence staining for SIV p28 (green), phospho‐ERK1/2 (yellow), and CCR5 (red) was performed on SIVE tissue (d). Unpaired *t* tests were performed for all statistical analysis. MPI analysis on a per‐cell basis was performed on 61 SIV p28− cells and 91 SIV p28+ cells showing a significant increase in phospho‐ERK1/2 MPI, *p*‐value < .0001 (e) and a significant decrease in CCR5 MPI, *p*‐value = .0013 (f). Only cells with above background CCR5 levels, > five adjusted MPI, were counted, while cells with a T‐cell morphology, small and very round, were excluded from this analysis. The ratio of phospho‐ERK1/2 to CCR5 was calculated for each cell showing a significant increase in SIV p28+ cells, *p*‐value < .0001 (g). Analysis of NanoString nCounter data for ERK1 (h) and ERK2 (i) showed a significant increase in ERK1 mRNA count, *p*‐value = .0256, but no change in ERK2 in animals with encephalitis.

Multiple studies have examined the endocytosis of CCR5 in vitro, presenting differing mechanisms that lead to either recycling of the receptor to the cell surface or sustained downregulation. Activated ERK1/2 signaling leading to clathrin‐mediated endocytosis has been shown to lead to sustained downregulation, with membrane expression following internalization dependent at least in part on de novo CCR5 synthesis (Venuti et al., 2015). To see if this mechanism persists in vivo, we first checked for the colocalization of clathrin heavy chain 1 with PVMs in SIVE tissue and observed strong colocalization (Figure 5a). The percentage of clathrin‐positive macrophages and MPI of clathrin‐positive macrophages was determined for UI, SIVnoE, and SIVE groups, with a significant difference in proportion between groups and significantly higher normalized MPI in SIVE animals (Figure 5b,c). We then examined phospho‐ERK1/2 as a measure of activated ERK1/2 signaling, and phospho‐ERK1/2 staining was present in cells with cytoplasmic CCR5. To further examine if phospho‐ERK1/2 contributed to the SIV‐associated CCR5 downregulation, MPI of phospho‐ERK1/2 was examined on SIV‐p28‐positive and SIV‐p28‐negative cells, and it was found that SIV‐p28‐positive cells express significantly more phospho‐ERK1/2 than uninfected cells (Figure 5d,e). Indeed, in SIVE animals, there was a significant increase in the ratio of phospho‐ERK1/2 to CCR5 in SIV p28‐positive cells (Figure 5g), indicating a conserved ERK1/2‐dependent mechanism of internalization that is accelerated in SIV‐infected cells. A study conducted by Venuti et al. (2015) found that specific ERK1 inhibition led to significantly less cytoplasmic CCR5 and β‐arrestin 1/2 accumulation, indicating inhibited internalization. To investigate potential differences in ERK1 versus ERK2, NanoString data of four UI and four SIVE frontal cortices were probed with the NHP Immunology V2 code set and examined for ERK1 and ERK2 mRNA expression levels in the brain. No difference was seen in ERK2 between uninfected and infected animals; however, there was a significant increase in ERK1 mRNA in SIVE‐infected animals supporting Venuti et al.’s findings (Figure 5h,i). This suggests that the increased internalization of CCR5 may be driven by increased ERK1 signaling.

## 4 DISCUSSION

The role of CCR5 in HIV pathogenesis as the major coreceptor of viral entry has been known in the field for over 25 years; however, its specific expression and importance in brain pathogenesis are as of yet unclear. In this study, we show an increase in total CCR5‐positive cells in the brain with progression to AIDS and encephalitis with infiltrating CD8 T cells representing the majority of these CCR5‐positive cells. Previous studies by our group have shown PVMs to represent the major cellular target of HIV and SIV and proliferation of PVMs during the development of SIVE (Filipowicz et al., 2016; Ko et al., 2019). In this study, we demonstrate for the first time, decreased expression of CCR5 in virally infected macrophages, which strongly suggests a virally driven CCR5 downregulation and a decrease in CCR5+ macrophages. Overall, we argue that increased activated ERK1/2 MAP kinase signaling seen here leads to an increase in clathrin‐mediated endocytosis of CCR5 and subsequent proteasomal degradation.

The significantly higher CD3 cell count in the brain of SIVE animals is in agreement with current literature on CD8 infiltrate in HIV/SIV infection, and the novel finding of high CCR5 expression on this infiltrate shown here indicates a substantial activated population (Subra & Trautmann, 2019). Studies by Fukada et al. (2002) and Wang et al. (2019) have shown all SIV/HIV specific tetramer‐positive CD8 T cells to be positive for CCR5 in PBMC's, lymph node, and jejunum in infected macaques and humans. These findings are in concurrence with previous studies of T cell populations in the brain of SIV‐infected macaques, which show an accumulation of antigen (virus)‐specific, activated CD8+ T cells in the brains of these animals (von Herrath et al., 1995; Marcondes et al., 2003). CCR5 expression on CD8 T cells is seen mainly on effector memory cells defined as CD45RA^−^CD28^+^CCR7^−^, with different groups reporting either negative (Wang et al., 2019) or lower expression of CCR5 on other subsets (Fukada et al., 2002), with no expression on naive cells. Despite this heightened specific CD8 response, the body is unable to clear infection or halt pathogenesis. T‐cell exhaustion in chronic infections due to over exposure to antigen has been shown in patients with HIV and may play a role in this inability to combat the infection (Li et al., 2021; Trautmann et al., 2006; Zhang et al., 2007). Further studies confirming phenotype of CCR5^+^ T cell subsets and examining exhaustion status on these cells are warranted.

In vitro CCR5 downregulation in response to receptor binding has been shown to be transient, with eventual re‐expression at the cell surface unless de novo synthesis of CCR5 is also inhibited (Mueller & Strange, 2004; Venuti et al., 2017, 2015). We demonstrate increased activated MAP kinase signaling in vivo, which leads to CCR5 internalization and degradation, in cells with high SIV p28 viral protein levels. HIV/SIV viral accessory protein nef is produced following the infection, and the expression of nef has been shown to downregulate multiple cellular proteins in a transcriptionally independent manner, including CCR5 (Dubey et al., 2008; Haller et al., 2014; Michel et al., 2005; Pawlak et al., 2018). Increasing expression levels of nef could induce continuous internalization and degradation of CCR5 in a ligand‐independent manor explaining the sustained downregulation observed. Alternatively, HIV/SIV infection has been shown to alter the transcriptional state of host cells to expedite viral pathogenesis and may block de novo synthesis of CCR5. Further work is needed to elucidate the full mechanism of sustained downregulation in PVMs. However, sustained decreased CCR5 surface expression on infected cells can facilitate the replication and preferential spread of virus to uninfected cells by avoiding viral re‐entry into infected cells, thereby promoting the pathogenesis of HIV/SIV.

CCR5 downregulation or agonist treatment has been explored as a potential therapy since its co‐receptor function was discovered. Two patients have now been hailed as “cured” of HIV after either bone marrow transplant or umbilical cord blood transplant from a ∆32/∆32 mutant CCR5 donor (Bone marrow transplant muffles HIV, 2009; Gupta et al., 2019). The ∆32 mutation causes a truncation in the CCR5 protein, preventing expression at the cell surface. Without adequate CCR5 expression, people carrying this mutation are naturally resistant to HIV infection (Ni et al., 2018). Following transplant, both patients have remained in remission without antiretrovirals for a minimum of 14 months or longer (Bone marrow transplant muffles HIV, 2009; Gupta et al., 2019). While these results are encouraging, the treatment and procedure is both dangerous and damaging to the recipients. Attempts to pharmacologically block CCR5 through antagonist treatment using maraviroc have shown reduction in viral replication and myeloid activation when applied soon after infection (Kelly et al., 2013). Maraviroc showed some success clinically, meeting non‐inferiority conditions in phase III clinical trials compared to then‐current ART and gaining approval by the FDA (Woollard & Kanmogne, 2015); however, clinical results have not fully lived up to promising preclinical investigations. Maraviroc has shown success halting the spread of infection; however, it likely has little to no effect on already infected cells with low to no levels of CCR5 expression (Tilton et al., 2010; Tiraboschi et al., 2017). This implies that maraviroc application will be most successful soon after infection, with reduced effectiveness in long standing infections. Combination therapy targeting CCR5‐low cells while simultaneously blocking spread to CCR5‐positive cells should be investigated.

## CONFLICT OF INTEREST STATEMENT

The authors declare no conflict of interest.

### PEER REVIEW

The peer review history for this article is available at https://publons.com/publon/10.1002/brb3.3126.

## Supporting information


**Supplemental FIGURE 1** PVMs exhibit surface and cytoplasmic CCR5 staining. Triple‐label immunofluorescence staining for CCR5 (green), CD163 (red), and DAPI (blue) shows surface staining (A,C) and cytoplasmic staining (B,D). Confocal Z plane analysis in (C,D) confirmed cytoplasmic localization of CCR5.
**Supplemental FIGURE 2** Percentage of CD3 T cells expressing CCR5. Triple‐label immunofluorescence staining for CCR5 (green), CD3 (red), and DAPI (blue) was performed and 50 CD3 T cells were counted as CCR5‐positive or ‐negative with one‐way ANOVA with Tukey's multiple comparison tests performed. The percentage of CD3+ lymphocytes expressing CCR5 increased significantly in SIVE animals in both vessel‐associated, p values = 0.0068 and 0.0133 (A) and total CD3 T cells, p values = 0.0355 and 0.0215 (B).

## Data Availability

The data that support the findings of this study are available from the corresponding author upon reasonable request.
