# Supplementary material for: Downregulation of CCR5 on brain perivascular macrophages in simian immunodeficiency virus‐infected rhesus macaques
Source: Brain Behav. 2023 Jun 27;13(8):e3126. doi: 10.1002/brb3.3126 (PMC10454275; doi:10.1002/brb3.3126)
Supplement: Supplementary file 1 — Supplemental FIGURE 1 PVMs exhibit surface and cytoplasmic CCR5 staining. Triple‐label immunofluorescence staining for CCR5 (green), CD163 (red), and DAPI (blue) shows surface staining (A,C) and cytoplasmic staining (B,D). Confocal Z plane analysis in (C,D) confirmed cytoplasmic localization of CCR5. Supplemental FIGURE 2 Percentage of CD3 T cells expressing CCR5. Triple‐label immunofluorescence staining for CCR5 (green), CD3 (red), and DAPI (blue) was performed and 50 CD3 T cells were counted as CCR5‐positive or ‐negative with one‐way ANOVA with Tukey's multiple comparison tests performed. The percentage of CD3+ lymphocytes expressing CCR5 increased significantly in SIVE animals in both vessel‐associated, p values = 0.0068 and 0.0133 (A) and total CD3 T cells, p values = 0.0355 and 0.0215 (B). [file BRB3-13-e3126-s001.docx]

**Downregulation of CCR5 on brain perivascular macrophages in simian immunodeficiency virus-infected rhesus macaques**

**Julian B. Hattler^1^, Derek L. Irons^1^, Jiangtao Luo^2^, Woong-Ki Kim^1,3,4^***

^1^Department of Microbiology and Molecular Cell Biology, Eastern Virginia Medical School, Norfolk, Virginia, USA; ^2^Department of Health Systems and Population Health Sciences, Tilman J. Fertitta Family College of Medicine, University of Houston, Houston, Texas, USA; ^3^Division of Microbiology, Tulane National Primate Research Center, Covington, Louisiana, USA; ^4^Department of Microbiology and Immunology, Tulane University School of Medicine, New Orleans, Louisiana, USA


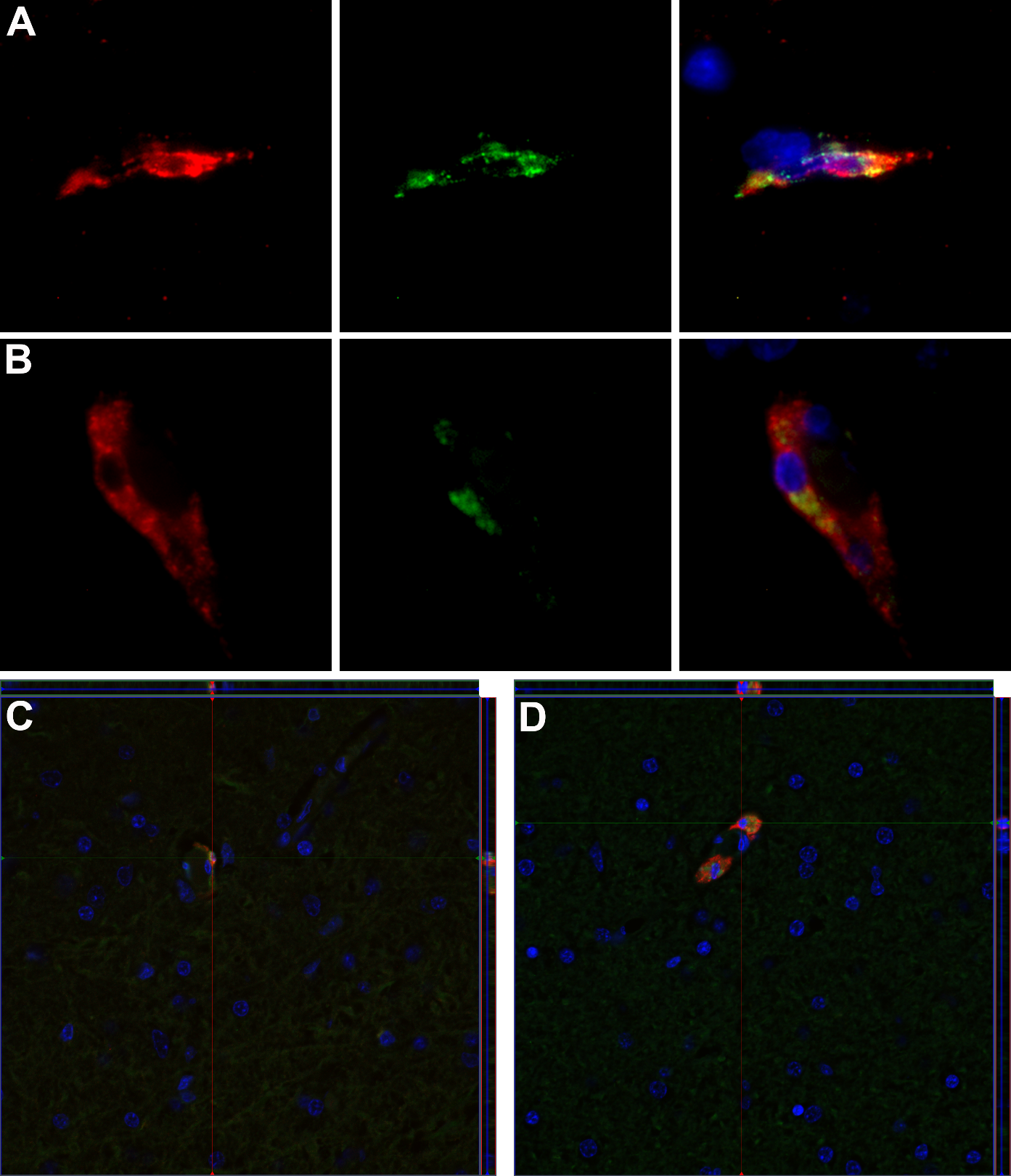


Supplemental FIGURE 1 PVMs exhibit surface and cytoplasmic CCR5 staining. Triple-label immunofluorescence staining for CCR5 (green), CD163 (red), and DAPI (blue) shows surface staining (A,C) and cytoplasmic staining (B,D). Confocal Z plane analysis in (C,D) confirmed cytoplasmic localization of CCR5.


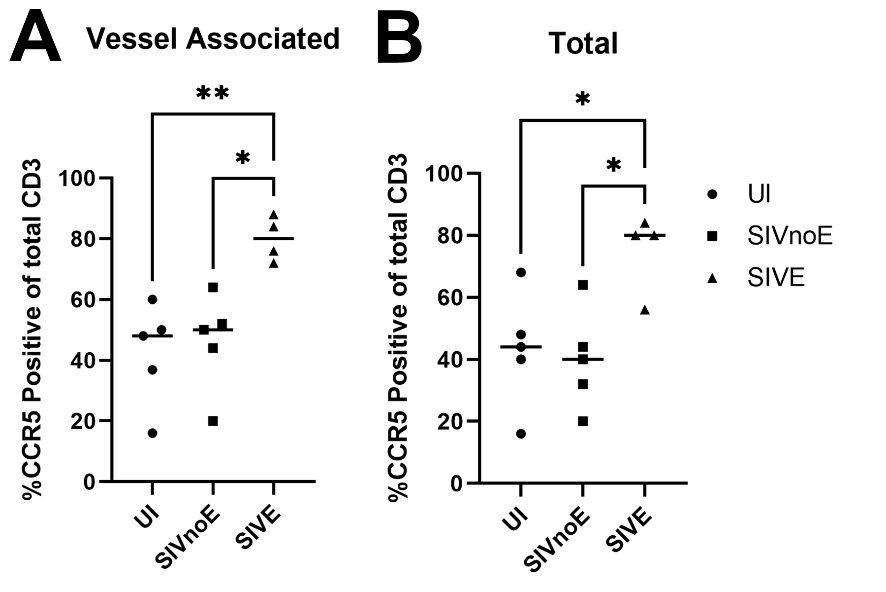


Supplemental FIGURE 2 Percentage of CD3 T cells expressing CCR5. Triple-label immunofluorescence staining for CCR5 (green), CD3 (red), and DAPI (blue) was performed and 50 CD3 T cells were counted as CCR5-positive or -negative with one-way ANOVA with Tukey’s multiple comparison tests performed. The percentage of CD3+ lymphocytes expressing CCR5 increased significantly in SIVE animals in both vessel-associated, p values = 0.0068 and 0.0133 (A) and total CD3 T cells, p values = 0.0355 and 0.0215 (B).
